# The functions of kinesin and kinesin-related proteins in eukaryotes

**DOI:** 10.1080/19336918.2020.1810939

**Published:** 2020-08-25

**Authors:** Iftikhar Ali, Wei-Cai Yang

**Affiliations:** a State Key Laboratory of Molecular Developmental Biology, Institute of Genetics and Developmental Biology, Chinese Academy of Sciences, Beijing, China; b The College of Advanced Agricultural Science, The University of Chinese Academy of Sciences, Beijing, China

**Keywords:** Kinesins, microtubule, motor domain, cargoes, cellular organelles

## Abstract

Kinesins constitute a superfamily of ATP-driven microtubule motor enzymes that convert the chemical energy of ATP hydrolysis into mechanical work along microtubule tracks. Kinesins are found in all eukaryotic organisms and are essential to all eukaryotic cells, involved in diverse cellular functions such as microtubule dynamics and morphogenesis, chromosome segregation, spindle formation and elongation and transport of organelles. In this review, we explore recently reported functions of kinesins in eukaryotes and compare their specific cargoes in both plant and animal kingdoms to understand the possible roles of uncharacterized motors in a kingdom based on their reported functions in other kingdoms.

## Introduction

Motor proteins, i.e. kinesin, myosin and dynein drive on cytoskeletal polymer microtubules, intermediate filaments and actin filaments, are key to diverse cellular activities like movement, division, transport as well as to maintain cellular shapes and mechanical integrity. In human, abnormalities in motor-driven transport have been linked to a wide range of diseases including cancer [1], polycystic kidney disease [2] and neurodegenerative diseases [3]. The molecular motors using the microtubule and actin cytoskeletons as their roads to move on and transport their attached cargoes [reviewed by 4], while these motor proteins do not move on intermediate filaments [5] but, rather, use them as their cargoes and thus help in their transportation. Microtubules (MTs) are tracks for the kinesin and dynein that move intermediate filaments, ribonucleoprotein particles and membrane-bound vesicles over long distances through the crowded cytoplasm [reviewed by 6]. These movements are responsible for the characteristic distributions of the Golgi apparatus, the endoplasm reticulum and other organelles in the cytoplasm. Microtubules also form the scaffold of the mitotic apparatus [reviewed by 7], and the axonemes of cilia and flagella [8]. In both cases, kinesins and dyneins act on the microtubule scaffold to move chromosomes during mitosis or to bend axonemes.

In plants, the microtubule is a central player in diverse environmental and developmental processes ranging from biotic and abiotic stress [9], tropisms [10], hormonal signaling [11], to cell expansion and division [12]. The structure and formation of cell walls are modulated by microtubules via influencing the structure and orientation of cellulose microfibrils within the cell wall, which then controls cell shape by bringing non-uniform cell enlargement in response to uniform intracellular turgor pressure. MTs are categorized into cortical microtubules residing in the cytoplasm in the form of cortical microtubules (CMTs) or lined with cell membrane, during the interphase stage of cell division. Cellulose microfibrils are added into the cell wall through the CMT-guided movement of membrane-associated cellulose synthase complexes. Furthermore, CMTs perform a variety of cell-specific configurations in order to accurately transmit intracellular signal to the extracellular matrix [13–15]. During mitosis, microtubules form the bipolar spindle that aligns sister chromatids in the middle of the cell before segregating them to opposite poles. Disruption of this mitotic apparatus can lead to aneuploidy, a major risk factor for birth defects, miscarriage, cancer development and other human diseases [16,17].

### Motor domain

Biochemical, genetic and molecular analyses of organisms from fungi to animals have discovered a common catalytic core region of about 350 amino acids with about 40% amino acid identity within the kinesin superfamily. This conserved catalytic region known as ‘motor or head’ domain is responsible for ATP hydrolysis and microtubule binding. The motor domain appears as a globular structure when visualized in the electron microscope [18], consists of a catalytic core mostly linked to a short region ‘neck linker’ that assists to bring the ATP-dependent conformational changes within the catalytic core and defines the direction of movement along a microtubule track [19].

**Figure 1.**
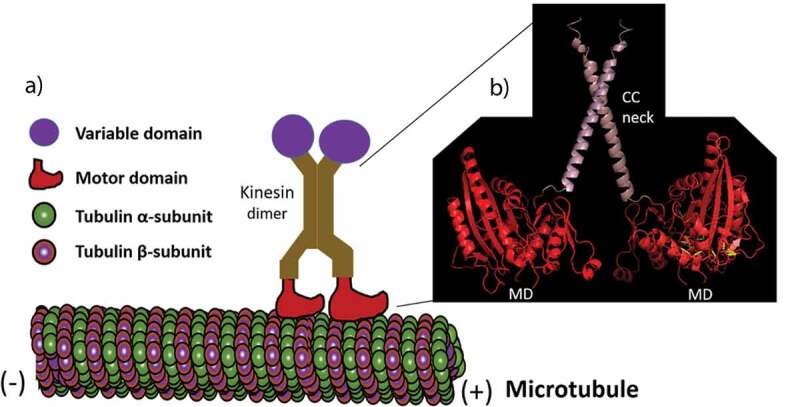
A kinesin walking on a microtubule. (a) A kinesin dimer using their motor domains to move on microtubules. (b) A ribbon diagram of the structure (made by I-TASSER online tool) of the motor domains (MD) and coiled-coil (CC) neck linkers of the *Arabidopsis thaliana* KCH kinesin.

Although 61 different kinesins encoding genes in a model plant *Arabidopsis thaliana* [20] have been identified with a high degree of similarity in their motor domains, the nonmotor sequences of these *A. thaliana* kinesins do not have common features. The nonmotor region consists of a ‘tail’ a highly variable domain found between different types of kinesins that is thought to mediate their binding to cargo [21]. The ‘head’ domain and the ‘tail’ domain are linked by a filamentous ‘stalk’ that consists of a coiled-coil domain (Figure 1; 20, 22). All members of kinesins have a domain with homology to the motor domain but little sequence match outside of this domain in the coiled-coil region. The tail domain, which is believed to interact with specific cargoes, is also not conserved among the kinesins.

The kinesins have been divided into 14 classes (Kinesin-1 to Kinesin-14) and some ungrouped orphans based on the phylogenetic analysis of their motor or catalytic domains [23]. However, the roles of most kinesins have not been characterized, the cellular activities in which they participate and the specific cargoes that they transport are also unknown. The functions of kinesin motors in mitosis have been reviewed already [24,25], here we focus on the important role of kinesins in diverse cellular processes in both plant and animal kingdoms.

### Plant kinesins

Kinesins have been well studied in animals, especially those from mice and humans. In contrast, only a few kinesins have been characterized in plants, mostly in a model plant *A. thaliana* [26,27]. Plants possess a large number of microtubule-based kinesin genes, for example, 78 kinesin genes were identified in the model moss *Physcomitrella patens*. However, these genes are not equally distributed to 14 identified families of kinesins; therefore, some of the kinesin families appear to have been lost in plants, while others have expanded and diversified extensively. For example, kinesin-2, 3, 9 and 11 families are absent from plants, while the Kinesin-7 and 14 families are greatly expanded with largest number of kinesin members. The reasons for the selective expansion of these kinesin families within the plant lineage are not clear. However, their abundance specifically in plants suggests that these kinesins have been evolved to achieve specified functions in plants including flower morphogenesis, trichome development, cell division and the formation of phragmoplasts [28,29] or to substitute the functions of those animal motors which are absent in plants like animal dyneins substituted by the motors of kinesin-14 family in plants.

### The functions of plant kinesins

**Figure 2.**
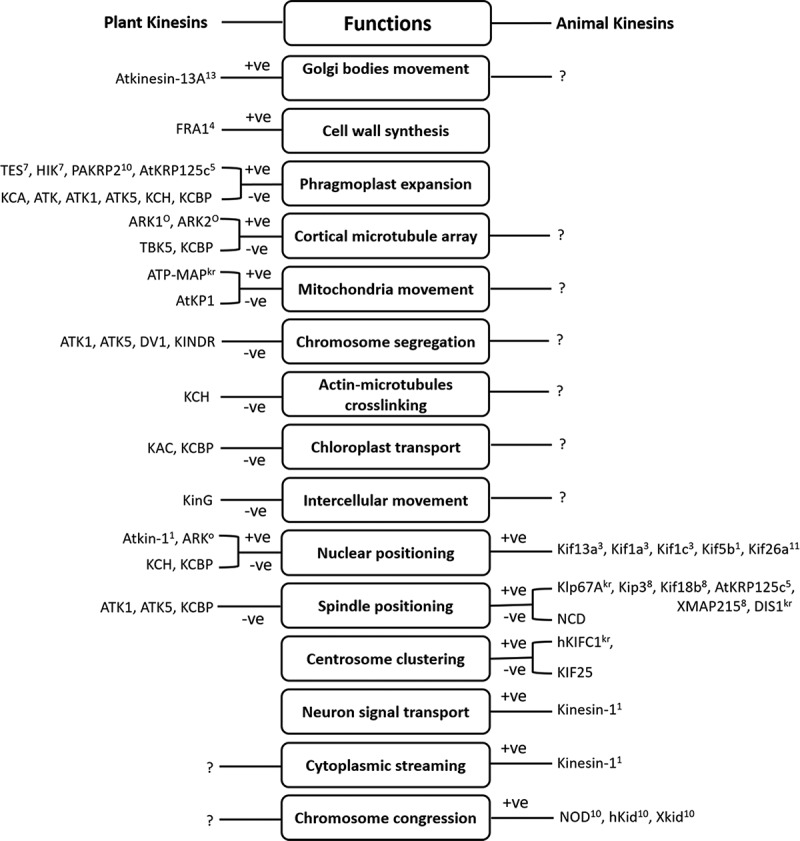
The role of kinesins and kinesin-related motors in diverse cellular activities. +ve: plus-end-directed motor, -ve: minus-end-directed motor, presence of superscript number: specific kinesin family (1–13), absence of superscript number: kinesin-14 family, O: orphan group kinesin, kr: kinesin-related proteins, symbol ?: existence of possibility for the similar kind of kinesins in the partner kingdom (plant or animal) which needs to be determined.

The correct spatial and temporal localization of organelles and molecules is essential for their function. Kinesin motors have been recognized to be involved in the local positioning of organelles and molecules by facilitating short-distance movements along microtubules [30]. While the long-distance movement of organelles and molecules is mostly believed to be driven by the actin–myosin interaction. Different kinesin motors are reported in the movement of several organelles, signifying specialization of kinesins for cargo transport (Figure 2). However, their roles in the long-distance movement of organelles/molecules in plants remain unresolved. Evidence is now collecting for kinesin-based motility of different organelles including Golgi bodies and Golgi-associated vesicles, chloroplasts, mitochondria and the nucleus.

### Phragmoplast expansion

The fundamental difference between plant and animal cell divisions is explained by the presence of a rigid cell wall in plants controlling the separation of two daughter cells. In contrast to animals, cytokinesis in plants is more complex and depends on the centrifugally expanding branching and bundled microtubules called phragmoplast that directs Golgi-derived vesicles to add cell wall ingredients between the two daughter cells [31]. Phragmoplast provides tracks for the delivery of Golgi-derived vesicles to the phragmoplast midzone during cell plate formation. Kinesins are imagined to be involved in the shipping of these vesicles. Several motors of kinesin-14 family, including kinesin-like calmodulin-binding protein (KCBP), kinesin interact with cyclin-dependent kinase CDK;1 (KCA) and *Arabidopsis thaliana* kinesin (ATK) in *P. patens* [32] and ATK1 [33], ATK5 [34], KCA1 and KCA2 [35], kinesin with calponin homology domain (KCH) [36] and KCBP [37] in the *A. thaliana* have been recognized to localize to the phragmoplast. The formation of phragmoplast is significantly delayed, either by reduction in the expression level of minus-end-directed ATK in *P. patens* [38] or by artificial activation of *A. thaliana* KCBP by blocking its self-inhibitory domain using antibodies [39]. KCA1 mutant fails to bind CDKA;1 and discourage depolymerization of phragmoplast microtubules [35]. In tobacco, NtKCH mediates phragmoplast expansion or organize phragmoplast poles by bundling microtubular minus‐ends [12].

In *A. thaliana*, kinesin-7 proteins TETRASPORE (TES) and HINKEL (HIK) are involved to disassemble microtubules in the center, when the phragmoplast expands outwards. Both HIK and TES shares about 57% amino acid sequence identity and may involve in cytokinesis of both male and female gametophytes [40,41]. The depolymerization of phragmoplast microtubules is promoted by the phosphorylation of microtubule associated protein 65 (MAP65), the target protein of *Arabidopsis* nucleus and phragmoplast-localized kinase1-related protein kinase 2 (ANP2), ANP3 and mitogen-activated protein kinase 4 (MPK4). Extensively bundled microtubules have been observed due to excessive under phosphorylated MAP65 in *anp2, anp3* and *mpk4* mutants [42]. HIK is localized at the phragmoplast midzone [43], binds and activates ANP1, 2 and 3 [44], supporting the view that kinesins are key to phragmoplast expansion by depolymerizing microtubules in the center through a mitogen-activated protein kinase (MAPK) pathway. Furthermore, phragmoplast-associated kinesin-related protein 2 (PAKRP2) in the kinesin-10 family has a motor domain in the N-terminus and is supposed to be a plus-end-directed motor, a necessary property for transporting vesicles to the phragmoplast midzone [45]. However, it remains unclear whether PAKRP2 is an active motor and whether it moves vesicles along phragmoplast microtubules or not.

Kinesin-5 motors may function in phragmoplast assembly by bundling and sliding overlapping microtubules at the midzone. Kinesin-5 family proteins in carrot and tobacco are localized to phragmoplast microtubules, especially at the plus ends [29,46]. Furthermore, mutant of the *A. thaliana* kinesin 5-c (AtKRP125c) in Kinesin-5 family shows severe phragmoplast defects with enlarged cells, multiple nuclei and incomplete cell plate deposition [47].

Phragmoplast-associated kinesin-related protein 1 (PAKRP1) and PAKRP1L in the kinesin-12 group are assumed to be involved in the phragmoplast formation by escaping the plus ends of the opposing microtubule sets from crossing the midzone and thus may determine the length of the overlapping region in the phragmoplast, and regulate microtubule dynamics [48]. When both kinesins are absent at the same time, the phragmoplast fails to assemble normally, resulting in defective cell plate formation. However, no clear defect has been observed for single mutants of these kinesins showing that they are likely to have redundant functions in the phragmoplast [48]. Moreover, the specific localization of these kinesins to the midzone of the phragmoplast where the microtubule plus ends of both halves of the phragmoplast face each other further increases our understanding for the involvement of these kinesins in the phragmoplast formation [49].

### Spindle positioning

Proper spindle placement is also particularly important to ensure accurate chromosome partitioning and symmetrical cell division. Hence, spindle positioning and orientation are critical during development and in stem-cell homeostasis, when cells divide asymmetrically to specify daughter cell fates and cell sizes [50]. The position of the spindle is influenced by the length and density of astral microtubules through modifying the interactions between cortical force generators and astral microtubules [51–53]. At the interphase-to-mitosis transition, the microtubules undergo rapid remodeling, allowing the depolymerization of long interphase microtubules followed by the assembly of astral microtubules and dynamic spindle that mark and position the bipolar spindle [54]. Kinesin-8 and kinesin-13 motors regulate microtubule dynamics and length across eukaryotes. In animals, the kinesin-13 proteins are known to regulate spindle microtubule dynamics and generate poleward microtubule flux, which is important for chromosome segregation [55]. However, the function of Kinesin-13 in plants is largely unknown.

In *Drosophila melanogaster*, kinesin-8 KLP67A (kinesin-like protein 67A) localizes to kinetochores, where it regulates spindle length [56], whereas kinesin-8 of budding yeast kinesin-related proteins 3 (KIP3) walks along microtubules and depolymerizes them [57]. Kinesin family member 18b (KIF18b) in the Kinesin-8 family is a highly processive plus-end-directed motor that uses its motile properties to reach microtubule ends, where it regulates astral microtubule length to ensure spindle centering. More recently, using *in vitro* reconstitution, the phosphorylation of C-terminal nonmotor microtubule-binding region was found to regulate KIF18b accumulation at plus ends which control KIF18b-dependent spindle positioning and regulation of microtubule length [58].

Kinesin-14 family proteins function in two ways in the spindle: firstly, cross-linking of antiparallel MTs near the spindle midzone and sliding them together, thus producing inward forces between spindle halves to equalize the outward forces created by Kinesin-5 motors, and secondly, cross-linking of parallel MTs in each half-spindle, where they focus minus ends into poles [59].

In animals, Kinesin-4 and 10 families functionin chromosome alignment, spindle organization and chromosome condensation [60,61]. They act as chromokinesins but which kinesins behave as chromokinesins in plants are largely unknown. Kinesin-7 family mediate kinetochore capture which is a basic function of spindle microtubules [61]. However, which kinesins achieve this activity in plants is also a question. Similar to that in animals and fungi, AtKRP125c in the Kinesin-5 localizes to the spindle and mutation of AtKRP125c results in fragmented or abnormal monopolar spindles [47]. *Xenopus* microtubule-associated protein 215 (XMAP215) promotes MT assembly at the plus end in *Xenopus* eggs [62] and defective in sister chromatid disjoining (DIS1) are critical for spindle function in yeast [63]. Furthermore, the kinesins localize to the spindle also include ATK1 and ATK5 in *A. thaliana* [28], and non-claret disjunctional kinesin-like protein (NCD) of kinesin-14 in *Drosophila* [64], suggesting the involvement of these kinesins to perform key steps in spindle positioning and orientation.

### Interphase cortical microtubule array

Plant cells generate highly organized microtubule arrays that coordinate expansion, cytokinesis and mitosis. In somatic plant cells, usually four alternating microtubule arrays are built regardless of division symmetry. Three successive MT arrays govern processes during cell division. The phragmoplast array partitions the cytoplasm, the spindle array segregates the duplicated chromosomes, and preprophase band (PPB) at G2 phase marks the future division site. While the cortical MTs contribute in directional expansion for cell morphogenesis during interphase [65,66]. On the other hand, MT arrays in pollen development possess some unique features, such as the assembly of curved phragmoplast MT array and an asymmetric spindle and the absence of the PPB to forecast the division plane [67].

In tobacco, TBK5 (kinesin-like protein expressed in tobacco BY-2 cells) in the Kinesin-14 family is assumed to function in gathering and relocating newly formed microtubules and microtubule-nucleating units. The CMTs are lost in tobacco cells with transient overexpression of GFP-TBK5 fusion protein [68]. In *A. thaliana*, At5G27950 shares about 60% amino acid sequence identity with TBK5. It will be exciting to identify the function of TBK5 counterpart in the model plant *A. thaliana*. Armadillo-repeat kinesin1 (ARK1) and ARK2 kinesins are also significant in controlling CMT organization, perhaps by enlightening microtubule depolymerization. In *A. thaliana*, overexpression of Kinesin-13A results in partial fragmentation of CMTs [69], showing that this kinesin might depolymerize CMTs. Kinesin-like calmodulin-binding protein (KCBP) in Kinesin-14 family affects trichome branch initiation through local reorganization and transient stabilization of CMTs [70]. Both microtubule organization 1 (*mor1-1*) and *mor1-2* mutants disrupt cortical MT arrays in root cells under restrictive conditions, but show normal cytokinesis and mitosis [71]. In short, several kinesins have been localized to the CMT array where they possibly facilitate CMT bundling and/or determine CMT dynamics. However, it is not necessary that localization of a kinesin to the CMT array does essentially indicate its job there.

### Microtubules size is controlled by motor and MAPs collaboration

During cell division, the shape and dynamic properties of the microtubule experience a number of notable transitions. One of these transitions is the formation of relatively stable, midzone microtubules in anaphase from the highly dynamic, metaphase spindle microtubules. Midzone microtubules held chromosomes away from each other after segregation and provide a platform for cytokinesis factors. Midzone microtubule construction is determined by the combined action of kinesin motor proteins and microtubule bundling proteins, and Kinesin-4 and protein required for cytokinesis-1 (PRC1) have appeared as key actors in human cells [72,73]. The microtubule-associated protein PRC1 and motor protein Kinesin-4 work together to mark microtubule ends for addition into the midzone in a length-dependent manner [74].

Kinesin-4, a plus-end directed motor protein activity, determines the localization of PRC1 [75] and inhibits microtubule dynamics [76]. PRC1 localizes to the midzone, where it crosslinks antiparallel microtubules [77]. An open question is how Kinesin-4 and PRC1 target a subset of biochemically identical spindle microtubules and specify their fates as midzone microtubules. The authors explain that Kinesin-4-PRC1 complexes recognize the microtubule and bind and move processively to the plus end. They experience a traffic jam on the arrival of many complexes at the plus end, thus marking an end tag from which the Kinesin-4-PRC1 complexes can detach. As the arrival rate of new complexes exceeds their rate of dissociation, the end tag will grow long. As the end tag grows, these rates are altered so that the dissociation rate of complexes from the end tags becomes equal to the rate of arrival of Kinesin-4-PRC1 complexes at the plus end and thus the steady-state length of the end tag is achieved [78].

### Chromosome segregation

Both mitotic and meiotic Kinesin-14s in plants are contributing to chromosome segregation and spindle pole including ATK1, ATK5, divergent spindle-1 (DV1) and kinesin driver (KINDR). In maize, KINDR in Kinesin-14 family localizes to neocentromeres *in vivo*, where it probably delivers the motile force for neocentromere activity and governs ATP-dependent minus-end-directed motility *in vitro*. KINDR is encoded by a cluster of genes on maize abnormal chromosome 10 (Ab10) that determine chromosome segregation in a process called meiotic drive. KINDR is involved in the motility of heterochromatic knobs during meiotic anaphase to alter chromosome segregation [79].

In plants, ATK of Kinesin-14 family influence chromosome alignment and segregation inhibition by affecting spindle architecture [80]. The major power to segregate chromosomes at anaphase does not come by the activity of molecular motor but created by the natural flux of tubulin from the plus ends (at kinetochores) to the minus ends (at poles) [81]. Furthermore, both Kinesin-7 and 13 families also regulate activation of the spindle checkpoint and kinetochore-microtubule attachment [82].

### Intercellular movement

Intercellular communication in plants is primarily carried out by cell-to-cell movement of transcription factors [83–85]. Many of these movable transcription factors serve as positional signals that regulate several features of plant development, including root patterning [85], stomata differentiation [86], root hair formation [84,87], floral initiation [88,89], shoot apical meristem maintenance [90,91], and embryonic development [92]. In plants, plasmodesmata are extremely specialized channels that form cytoplasmic continuity for the intercellular movement of molecules and thus permit the exchange of proteins between two adjacent cells [93]. Both viral movement proteins and endogenous plant proteins interact with microtubules to regulate their movement through plasmodesmata. The virus-associated replication complexes are synthesized by the association of microtubules with viral movement proteins, which are essential for the amplification and subsequent spread of the virus. In contrast, it remains less clear how the microtubule activity regulates the intercellular movement of plant proteins. However, the well-known example in plant is Kinesin G (KinG; At1G63640) in Kinesin-14 family interacts with short-root (SHR) protein and promotes its movement between cells in the root to regulate root radial patterning. The association of SHR with KinG regulates the synthesis of stable movement complexes that assist the cell-to-cell transport of SHR [94]. SHR movement is vital for the asymmetric divisions of the cortical endodermal daughter cells that produce the distinct layers of endodermis and cortex [95]. Analysis of KinG and SHR dynamics and localization in live cells proposes that KinG is a nonmotile motor that encourages the pausing of SHR-associated endosomes. Furthermore, SHR movement is greatly reduced with loss of function mutant of *king* [94], suggesting the potential role of KinG in the intercellular movement of proteins.

### Chloroplast transport

Photosynthesis is an important process on which all living systems depend, is carried out in autotrophic organism in a plant cell-specific organelle, a chloroplast. As any change in external light condition is detected, photosynthetic cells adjust this change by redistribution of chloroplasts to disperse it in intense light or concentrate it in low light in the direction of the light source [96]. Most of the studies support that actin filaments rather than microtubules are involved in chloroplast movement because of the construction of short actin filaments, termed chloroplast-actin filaments, around the chloroplasts [97–99]. As myosins, major actin-binding motor proteins are hypothesized to be involved in the process, but the lack of evidences in plants for myosin contribution making the question more complicated [100,101]. Loss-of-function mutants of myosin XIs in tobacco as well as in model plant *A. thaliana* does not exhibit any defects in chloroplast movements [reviewed in 102]. Minus-end-directed motor proteins in Kinesin-14 family that binds both actin and microtubule-based cytoskeletal elements are suggested to have potential functions in chloroplast positioning. *In vivo* experiments discovered that chloroplasts are unevenly dispersed and assembled toward the growing ends, with minus-end directionality [38]. Microtubule plus ends directionality with abnormal chloroplast distribution has been observed for KCBP knockout mutant in *P. patent*, showing the important role of kinesin in chloroplast positioning [38]. Another C-terminus minus-end-directed motor protein is Kinesin-like protein for actin-based chloroplast movement (KAC) that does not show microtubule-dependent motility *in vitro* but contains an actin-binding domain [103,104]. Both *kac1* and *kac2* mutants in *P. patens* and *A. thaliana* exhibited defects in chloroplast relocation and attachment to the plasma membrane [103–105]; however, it is not clear how KAC accomplishes its function in chloroplast relocation.

### Golgi bodies movement

In animals and fungi, Golgi body movement is carried out by microtubule-based, minus-end-directed dynein motors [106]. In plants, minus-end-directed Kinesin-14s are considered as the counterpart of dynein, but no Kinesin-14 has been reported yet to be involved in Golgi stack dispersion. However, a motor protein from Kinesin-13 family, Kinesin-13A has been shown critical in the distribution of Golgi bodies and the budding of Golgi-associated vesicles. The *kinesin-13a-1* loss-of-function mutant shows abnormal morphology of Golgi cisternae with fewer and smaller Golgi-associated vesicles in the peripheral cells [107]. The mutant also shows more branches in leaf trichomes and aggregation of Golgi stacks [108]. In both root-cap peripheral cells and leaf cells in *A. thaliana*, Kinesin-13A was found to be localized on Golgi-associated vesicles [107]. This data suggests that Kinesin-13A motor plays an important role in the structure and function of Golgi stacks and the formation of Golgi-derived vesicles; however, the mechanism for this function remains unclear.

### Mitochondria movement

Like Golgi stacks, the movement of mitochondria is also frequently regulated by dynein motors [109]. In plants, two kinesins have been found to regulate the function and/or motility of mitochondria. Kinesin-14 AtKP1 motors localize to mitochondria [110], specifically bind to the mitochondrial outer membrane protein voltage-dependent anion channel VDAC3 and regulate ATP levels and aerobic respiration during seed germination at low temperature [111]. In addition, in tobacco pollen tube, another motor protein was found to localize with mitochondria that might be important for the positioning and motility of mitochondria [112].

### Nuclear positioning

The nucleus is the central feature of eukaryotic cells and is often described as the largest organelle. Nuclei are actively positioned asymmetrically depending on cell differentiation status, migratory state, cell cycle and also cell type. For example, in fertilized invertebrate and mammalian eggs, male and female pronuclei start movement toward each other and fuse near the middle of the zygote, satisfying that the resulted cell division generates two equal daughter blastomeres. Nuclei are frequently positioned in the middle of the fission yeast *Schizosaccharomyces pombe*, satisfying that the division plane builds two equal daughter cells. Furthermore, during cell division in budding yeast, nuclei are moved into the bud neck so that each daughter cell receives one [113]. Over the past decade, it has made clear that the nucleus is tightly assembled into the structural network of the cell through so-called linker of the nucleoskeleton and the cytoskeleton (LINC) complexes, which allow delivery of forces between the cytoskeleton and nucleus. This physical association between the cytoskeleton and the nucleus is critical for a broad range of cellular functions, including cell migration, cell polarization, cytoskeletal organization and intracellular nuclear movement and positioning. The LINC complex contains two families of proteins with KASH domain proteins present at the outer nuclear membrane and the SUN domain proteins present at the inner nuclear membrane, they interact with each other through their KASH and SUN domains across the luminal space. KASH domain proteins can bind to all major cytoskeletal filament networks, including microtubules (via dynein and kinesin motor proteins binding to KASH5, nesprin-1, 2 and 4), intermediate filaments (via interaction of nesprin-3 with the cytoskeletal linker plectin), and actin filaments (through the actin-binding domain of the giant isoforms of nesprin-1 and −2). SUN domain proteins interact with the nuclear lamina, nuclear pore proteins, and other nuclear proteins at the nuclear interior; in the cytoplasm [113]. Here, the involvement of kinesin-based motors will be discussed in detail with respect to their roles in nuclear positioning and movement.

Nuclear positioning is a determining event in several cellular processes, such as cell differentiation, cell migration, and fertilization. During muscle development or regeneration, nuclei are migrated to the periphery of mature skeletal myofibers which are normally present in the center of myotubes [114]. The correct positioning of each individual nucleus during myofiber development requires multiple nuclear movement events [115-117,118]. Proper nuclear positioning has been found a key in the function and structure of muscle cells [117], and nuclear mispositioning is an indication of different muscle disorders, such as centronuclear myopathies [119].

During muscle differentiation, nuclear movements along the myotube axis might represent the event required for the even positioning of nuclei in the mature myofiber. Actin, as well as microtubule cytoskeleton, is involved in nuclear positioning [113]. Recently, microtubule-related motors have been studied in nuclear behavior, alignment, speed and time in motion during myotube differentiation. About 19 different kinesins including KIF1a, KIF1c, KIF5b, KIF9 and KIF21b affect nuclear movement, alignment and behavior by microtubule-dependent mechanisms [120]. Even though recent advances in understanding nuclear positioning in the development of muscle and brain cells demonstrate a central role of some microtubules-related motors such as dyneins and kinesins [115, 117], the contribution of other MT motors to nuclear positioning during myofiber formation remains unexplored.

In the moss *P. patens*, a knockout mutant of *KCBP* exhibited defect in the nuclear movement after cell division, in which the daughter nuclei stayed at the cell plate and did not move to the cell centers [38]. Furthermore, KCBP localizes to the nucleus beginning in prophase and persists until nuclear envelope breakdown in the dividing cotton cells [121], further signifying that KCBP is involved in nuclear migration. AtKin-1, the Kinesin-1 member of *A. thaliana*, plays a role during female gametogenesis through the regulation of nuclear division cycles [122]. In *A. thaliana, Gossypium hirsutum, Nicotiana tabacum* and *Oryza sativa*, KCH motors localize to perinuclear actin filaments that surround the nucleus and position it to the cell periphery [12,123]. In rice, OsKCH1 is thought to regulate nuclear migration by building a coordination between actin and microtubule cytoskeleton activities and its overexpression delay nuclear positioning and the onset of mitosis, although it still proceeds through mitosis normally [123]. However, it is still unresolved whether the regulation of nuclear migration by KCH kinesin is conserved in plants. OsKCH1 shares about 50% amino acid sequence identity with *A. thaliana* At2G47500. It will be exciting to check whether *A. thaliana* protein exhibits similar role in nuclear movement.

Nuclear positioning requires a tug-of-war between minus-end and plus-end-directed kinesin motors. In animals, this task requires plus-end directed kinesins and minus-end-directed dyneins. For example, KIF1a in the Kinesin-3 family has been implicated for plus-end directed movement and dynein for minus-end-directed movement to control nuclear positioning [124]. As plants lack the major minus-end-directed dynein motors for cargo delivery, however they contain calponin homology domain (KCH) subclade of the Kinesin-14 family to substitute the function of dynein in animals. The loss of function mutants of plus-end-directed ARK kinesins triggers basal nuclear accumulation near the newly formed cell plate [125]. However, more recently the first plant lacking KCH is obtained through deletion of all four *KCH* genes in moss which showed completely opposite phenotype, the accumulation of nuclei at the apex rather than gravitating to the center of tip growing protonemal cells during interphase [126]. KCH kinesins move processively *in vivo* as a minus-end-directed motor shown by KCH fluorescent-tagging experiments. Hence, it seems that nuclear positioning involves a tug-of-war between the KCH, a minus-end-directed motor, and ARK, a plus-end-directed motor, an imbalance of which consequences in abnormal nuclear invaginations and nuclear distribution skewed toward the cell periphery [127]. Finally, in plants, the involvement of Kinesin-14 in the nuclear migration during cell division suggests a potential substitute for the function normally done by dynein.

### Cell wall synthesis

In differentiated cells, such as xylem and interfascicular fibers, the thick secondary cell walls provide strength to withstand large negative pressures and gravity. While in growing cells, the tough but extensible primary wall determines the direction and rate of expansion and overall plant shape. Cell wall consists primarily of pectin, hemicellulose and cellulose along with small amounts of proteins. Cellulose microfibrils are synthesized *de novo* at the plasma membrane, while pectin and hemicellulose are manufactured in the Golgi and then transported to the extracellular space through the secretory system. Cellulose microfibrils are synthesized by cellulose synthase complexes (CSCs) which are thought to be assembled in the Golgi and trafficked to the plasma membrane [128]. The CSCs are an important constituent of the secretory vesicles that facilitate growth in diffusely growing cells. CSCs trafficking depends on long-distance myosin motor’s drive on actin filaments [129], without which CSCs can reach the plasma membrane but at a reduced rate [130]. Furthermore, cortical microtubule arrays also have critical roles in cell wall development. Electron micrographs show that CMTs occur in the vicinity of the plasma membrane, with a constant distance from the membrane [131], linking the PM and MT by cross bridges [132]. The presence of cross bridges hypothesizes that PM may be anchored to the CMTs by some linkers. In angiosperm, the transverse CMT array is detected in most interphase bipolar cells except for highly specialized cells such as dead xylem cells and tip growing cells. The CMTs are also associated with several endomembrane systems to regulate cell wall development and other cellular events [133]. In short, the presence of highly ordered CMTs tightly anchored to the plasma membrane guides the deposition pattern of cellulose microfibrils by regulating the movement of the cellulose synthase complex.

The FRAGILE FIBER1 (FRA1) in the Kinesin-4 family was first identified as a regulator of cellulose microfibril alignment in secondary cell walls [134,135]. However, recent work shows that FRA1 is a processive motor [136], and regulates vesicle trafficking of non-cellulosic components along CMTs in both primary and secondary cell walls [137,138]. Live imaging of the fluorescently tagged FRA1 discovered that FRA1 is localized to particles that move along CMTs at much faster velocities than CSCs. The *fra1* null mutant displays a slightly reduced pectin content with reduced cell wall thickness. However, the *fra1* plants display little defects in CSC velocity, amount of cellulose microfibrils, CMT alignment and organization, hypothesizing that FRA1 is not critical in cellulose microfibril synthesis [137,138]. Another motor KCBP in Kinesin-14 family which serves as cargoes of chloroplasts and nuclei along MTs in the *P. patens* [27,38] may also regulate cell wall development. KCBP is localized at the tip of growing branches, and loss of KCBP causes deformation of CMTs [139], supporting the hypothesis that KCBP guides cell wall development by regulating CMT organization.

### Functions of animal kinesins

#### Neuron signal transport

Neurons depend on the polarized distribution of organelles, macromolecules and vesicles in axons and dendrites for a continued network of sending and receiving signals. The majority of transport in neurons is facilitated by dynein and kinesin motors driving on the microtubule and trafficking the cargoes in both directions. The proper delivery of both incoming and outgoing cargoes to their proper destinations in the cell body needs tightly controlled kinesin-driven events. In invertebrate neurons, dendrites contain minus-end-distal MTs with dynein motors whereas axons contain predominantly plus-end-distal MTs with kinesin motors such as Kinesin-1 [140,141]. In contrast, mammalian neurons contain plus-end-distal MTs in both dendrites and axons suggesting that Kinesin-1 must be highly regulated to prevent kinesin-driven transport in dendrites, and to distinguish between dendritic and axonal MTs [142–144], to achieve compartment-specific cargo distribution. However, it remains unclear how Kinesin-1 activity is planned to maintain the compartment-specific localization of cargo. Recently, it was proposed that Kinesin-1 activity is accurately regulated by autoinhibition to accomplish the selective localization of dendritic cargo [145].

#### Centrosome clustering

In human, a minus-end-directed motor KIFC1 in the Kinesin-14 family is known for its centrosome clustering in multi-centrosome cells and managing multi-polar spindles into pseudo-bipolar spindles to avoid multi-polar division [146]. KIFC1 is strongly expressed in cancer cells and result in numerous cancer types including testis cancer in man [147,148]. Furthermore, KIFC1 motor plays important roles in cancer cell division, which usually possesses more than two centrosomes and thus measured to be a new-generation chemotherapy target for cancer [149,150]. However, the importance of KIFC1 in normal cell types remains unclear.

#### Chromosome congression

Genomic stability in organisms is maintained by the equal distribution of duplicated DNA during cell division. The stable transmission of the genome to daughter cells during cell division relies on the process of chromosomal positioning at the spindle equator known as chromosome congression. Microtubules plus-end directed motor proteins called chromokinesins, facilitate congression. Kinesin-4, 10, and 12 have been recognized to behave as chromokinesins, function together to promote chromosome alignment during cell division [151–153].

A nonmotile, orphan kinesin no distributive disjunction (NOD) is identified as chromokinesin in *Drosophila melanogaster* which contributes to congression. NOD directly binds EB1 through unconventional EB1-interacting motifs that are similar to a newly characterized MT tip localization sequence [154]. High-resolution imaging of EB1 and NOD-coated chromatin stretching events in living cells suggest both plus-end-directed motility and end-tracking [155]. NOD moves chromosomes by associating with the plus ends of polymerizing MTs [156,157], although direct evidence that how could a nonmotile kinesin produce force to accomplish this mechanism is lacking. NOD was first recognized as the mutant *no distributive disjunction* (*nod*), which displayed high frequencies of chromosome loss and nondisjunction in female meiosis [158,159]. It has been hypothesized that NOD supports congression, not by conventional plus-end-directed motility, but by accelerating polymerization forces by end-tracking on growing MT plus ends via a mechanism that is also unclear.

Furthermore, vertebrate Kinesin-10s have been shown to possess plus-end-directed motility and to create force when associated with chromatin [72,152,160]. Kinesin-10, human kinesin-like DNA binding protein (hKID) identified in human contributes to establish and maintain chromosome congression [152,153,161], while the severe alignment defects were observed in xKID in egg extracts from *Xenopus laevis* [162,163].

#### Cytoplasmic streaming

Transport processes that purposefully move chromosomes, organelles, and other objects from one place to another through cytoplasm are critical to cell development, growth and reproduction. Cytoplasmic streaming existed in most cells of angiosperms is the easily detectable movement as the rapid motions of refractive particles inside the cell. This type of movement is known as the standard example of long-distance transport in plant cells first reported for *Charophycean* algae in the eighteenth century [164]. In the model plant *A. thaliana*, reduced movement speeds of different organelles have been observed with the loss of single or multiple myosin XI motors which move specifically on actin cytoskeleton [165,166], suggesting that cytoplasmic streaming is frequently driven by myosin [167]. Many individual organelle movements have been detected during cytoplasmic streaming by using fluorescent markers [168,169]. In plants, kinesins based cytoplasmic streaming is not noticed yet, however, microtubule-based kinesins fluid streaming has been identified in animals. For example, the germline stem cell in the *Drosophila* ovary produces an egg chamber with 15 nurse cells that are connected by cytoplasmic streaming to one another and to the anterior end of a developing oocyte. A plus-end-directed microtubule motor, Kinesin-1 is involved to generate force for this cytoplasmic streaming [170]. However, despite many explanations of cytoplasmic streaming over the years, the exact nature of the flow is still not fully resolved.

## Conclusions

Subcellular localization of kinesins has provided great insights into the cellular functions of these motors through live-cell imaging or immunofluorescence microscopy. Kinesin family proteins are found throughout eukaryotes and show predominantly splendid localization patterns from chromosomes and kinetochores to MT arrays like the phragmoplast midzone, phragmoplast distal ends, the spindle midzone, spindle poles, the preprophase band and the cellular organelles. Minus-end-directed kinesins are mostly involved in the transport of big cargoes (substitute function of dynein) while small cellular particles are mainly drive by plus-end-directed motors. Diverse subcellular localizations conclude undefined jobs of these motors in critical cellular pathways that need to be characterized further.
